# Dietary Antioxidants and Nrf2-Related Redox Responses in Sheep: Evidence, Limitations, and Implications for Health and Productivity

**DOI:** 10.3390/vetsci13090875

**Published:** 2026-08-27

**Authors:** Shahab Ur Rehman, Aftab Shaukat, Mohamed Tharwat, Asfand Yar Khan, Abdulrahman A. Alkheraif, Rahmat Ali

**Affiliations:** 1 Laboratory of Metabolic Manipulation of Herbivorous Animal Nutrition, College of Animal Science and Technology, Yangzhou University, Yangzhou 225009, China; shahab@yzu.edu.cn; 2 Animal Science Division, Department of Modern Agriculture, College of Life and Environmental Sciences, Hunan University of Arts and Science, Changde 415000, China; dr.aftabshaukat@huas.edu.cn; 3 College of Veterinary Medicine, South China Agricultural University, Guangzhou 510642, China; 4 Department of Clinical Sciences, College of Veterinary Medicine, Qassim University, P.O. Box 6622, Buraidah 51452, Saudi Arabia; atieh@qu.edu.sa; 5 Department of Clinical Sciences, Faculty of Veterinary Sciences, The University of Veterinary and Animal Sciences, Swat 19120, Pakistan; dr.asfandkhan888@gmail.com; 6 Department of Pathology and Laboratory Diagnosis, College of Veterinary Medicine, Qassim University, P.O. Box 6622, Buraidah 51452, Saudi Arabia

**Keywords:** Nrf2, *Keap1*, antioxidant response element, oxidative stress, sheep, dietary antioxidants, polyphenols, tannins, selenium, evidence grading

## Abstract

Oxidative imbalance can accompany physiologically demanding and environmental transitions in sheep, but its magnitude varies with tissue, production stage, diet, health status, and the biomarkers used. Nuclear factor erythroid 2-related factor 2 (Nrf2; encoded by *NFE2L2*) coordinates inducible cytoprotective responses through Kelch-like ECH-associated protein 1 (Keap1), antioxidant response elements (*AREs*), and Keap1-independent regulatory routes. This structured narrative review evaluates whether dietary antioxidants engage Nrf2-related responses in sheep and whether such responses translate into health, productivity, or product-quality benefits. Evidence was classified by model and endpoint. Tier 1 comprised ovine dietary interventions with tissue-level Nrf2-pathway measurements; Tier 2 comprised ovine dietary studies with redox or phenotypic outcomes but no pathway assay; Tiers 3 and 4 comprised other-ruminant and non-ruminant or in vitro mechanistic evidence, respectively. Only rutin in transition-period ewes and a water extract of *Artemisia annua* in lambs met Tier 1 criteria. Both altered Nrf2-related gene expression, but neither used pathway perturbation, DNA-binding assays, or definitive target-engagement methods; the results therefore indicate association rather than causality. Evidence for tannins, essential oils, vitamins, selenium, carotenoid-rich feeds, and other phytochemicals in sheep is broader for oxidative status and product stability than for Nrf2 activation. Growth, fertility, milk yield, and survival outcomes are heterogeneous and strongly context-dependent. Dietary antioxidants may act through direct radical interception, microbial metabolites, metal chelation, membrane protection, mitochondrial effects, receptor signaling, inflammation control, and, in some settings, Nrf2-related adaptation. Future studies should combine dose–response designs, exposure measurements, multiple redox markers, tissue-specific pathway assays, and causal validation. Accordingly, Nrf2 is a plausible mechanistic framework for sheep nutrition, but current evidence does not support broad causal or productivity claims.

## Simple Summary

Sheep encounter oxidative challenges during late gestation, lambing, early life, weaning, rapid growth, lactation, reproduction, disease, and heat exposure. The Nrf2–Keap1–antioxidant response element (ARE) system helps cells adapt by regulating antioxidant, detoxification, and repair processes. Dietary antioxidants may support this system, but the sheep-specific evidence must be interpreted cautiously. Only two dietary studies in sheep have measured Nrf2-related molecular readouts directly, and both show pathway-associated changes rather than causal proof of Nrf2 activation. Most other sheep studies report antioxidant-enzyme activities, oxidative-damage markers, health outcomes, or product quality without measuring the pathway. This review separates those evidence levels, summarizes practical benefits and limitations, and identifies the experiments needed before Nrf2-targeted feeding can be recommended reliably. Current evidence supports context-specific antioxidant nutrition, particularly during documented oxidative challenge, but not the assumption that every antioxidant supplement works through Nrf2 or improves productivity.

## 1. Introduction

Reactive oxygen species (ROS) are produced by mitochondrial respiration, NADPH oxidases, xanthine oxidase, endoplasmic-reticulum protein folding, peroxisomal reactions, and the respiratory burst of immune cells. At controlled concentrations, particularly in defined subcellular compartments, ROS participate in signaling, host defense, and metabolic adaptation [1,2]. Oxidative stress is therefore not simply the presence of ROS; it is a disruption of redox signaling and control in which oxidant generation exceeds the location- and time-dependent capacity of buffering, repair, and adaptive systems. Persistent imbalance can damage lipids, proteins, and nucleic acids and can accompany inflammation and impaired animal performance [2,3,4,5].

Sheep may experience such imbalance during late gestation and early lactation, birth, weaning, rapid growth, reproduction, disease, transport, and heat exposure. These periods differ in cause, tissue involvement, duration, and severity, so no single biomarker or feeding strategy can represent all of them. Vitamin E and selenium have long been used to support antioxidant function in ruminants, and plant-derived polyphenols, tannins, essential oils, algae, and agro-industrial by-products are increasingly studied [4,6,7,8,9]. Their effects may involve direct chemical antioxidant action, altered rumen fermentation, microbial metabolites, membrane stabilization, mitochondrial or receptor-mediated responses, control of inflammation, and adaptive transcriptional signaling.

The Nrf2–Keap1–ARE pathway is a major regulator of inducible cytoprotection [10,11,12,13,14]. However, higher superoxide dismutase (SOD), catalase (CAT), glutathione peroxidase (GPx), or total antioxidant capacity (TAC), or lower malondialdehyde (MDA), does not by itself demonstrate Nrf2 activation. Direct pathway claims require appropriate tissue measurements and ideally nuclear localization, *ARE* binding or reporter activity, target-gene and protein responses, or perturbation of Nrf2/Keap1 function. This distinction is especially important in sheep because most mechanistic evidence comes from non-ovine models, whereas ovine studies usually report downstream redox or performance endpoints.

Accordingly, this review asks three questions: (i) what constitutes credible evidence of Nrf2 pathway engagement in sheep; (ii) which dietary antioxidant classes improve redox, health, reproductive, or product-quality outcomes in ovine studies; and (iii) what dose, safety, bioavailability, and experimental limitations prevent translation into feeding recommendations. Knowledge gaps—including the scarcity of tissue-specific and causal ovine evidence—are stated here and revisited in Section 7.

### Review Approach and Evidence Classification

This article was prepared as a structured narrative review rather than a systematic review or meta-analysis. The original reference set was checked and updated through targeted searches of PubMed, Crossref, reference lists, and publisher websites, with the last search performed in August 2026. The main publication window was January 2000 to August 2026, although earlier seminal mechanistic work was eligible when needed. Search concepts combined sheep, ewe, lamb, or ovine with *Nrf2*, *NFE2L2*, *Keap1*, *ARE*, oxidative stress, redox, antioxidant, polyphenol, flavonoid, tannin, essential oil, vitamin E, selenium, carotenoid, algae, heat stress, transition period, rumen, mammary gland, meat, milk, sperm, embryo, fertility, growth, or feed efficiency. English-language peer-reviewed full papers, reviews, and meta-analyses relevant to the scope were considered. Conference abstracts, duplicate reports, studies without a relevant redox or production endpoint, and sources whose model or intervention could not be verified were excluded.

Evidence was prioritized in the following order: controlled ovine feeding interventions; ovine observational or ex vivo studies; small-ruminant or other-ruminant evidence; and non-ruminant animal or cell models used only to explain mechanisms not yet tested in sheep. Tier 1 denotes an ovine dietary intervention that measured *Nrf2*, *Keap1*, *ARE* activity, or recognized pathway-related genes/proteins in tissue. Tier 2 denotes an ovine dietary intervention with redox, health, productivity, or product-quality endpoints but no Nrf2-pathway measurement. Tier 3 denotes other-ruminant evidence. Tier 4 denotes non-ruminant or in vitro mechanistic evidence. Tier 1 indicates pathway association, not causality, unless the study also demonstrates nuclear translocation or *ARE* engagement and uses an Nrf2-specific perturbation or equivalent target-validation strategy. Because designs and endpoints were heterogeneous, evidence was synthesized narratively, and no pooled effect estimate was generated.

## 2. Oxidative Stress and Redox Homeostasis in Sheep

### 2.1. Sources, Compartments, and Buffering Networks

Redox homeostasis is dynamic and compartment-specific. Mitochondria, peroxisomes, the endoplasmic reticulum, plasma membranes, and activated leukocytes generate different reactive species at different rates. Cytosolic and mitochondrial pools of NADPH, reduced glutathione (GSH), thioredoxin, and peroxiredoxins buffer these signals and support repair. SOD converts superoxide to hydrogen peroxide; CAT, GPx, and peroxiredoxins then reduce hydrogen peroxide or lipid hydroperoxides. Glutathione reductase (GSR) and thioredoxin reductases use NADPH to restore reducing capacity [1,2,15]. Selenium is required for several GPx and thioredoxin-reductase enzymes, while copper, zinc, and manganese support SOD isoforms [15,16].

Low-level ROS signaling is physiologically useful, so the goal of nutrition is not maximal suppression. Excessive reducing pressure can produce reductive stress, defined as a pathological shift toward overly reduced cellular redox couples that disrupts disulfide-bond formation, signaling, protein quality control, and mitochondrial function [17]. Thus, both oxidative and reductive extremes can be harmful. Antioxidant responses may also be biphasic: a low exposure may induce adaptive defenses, whereas a high exposure may be ineffective, pro-oxidant, cytotoxic, or antinutritional. This hormetic possibility is a reason to avoid assuming that a higher supplement dose is necessarily better [12].

### 2.2. Physiological and Environmental Windows of Redox Challenge

**Figure 1:**
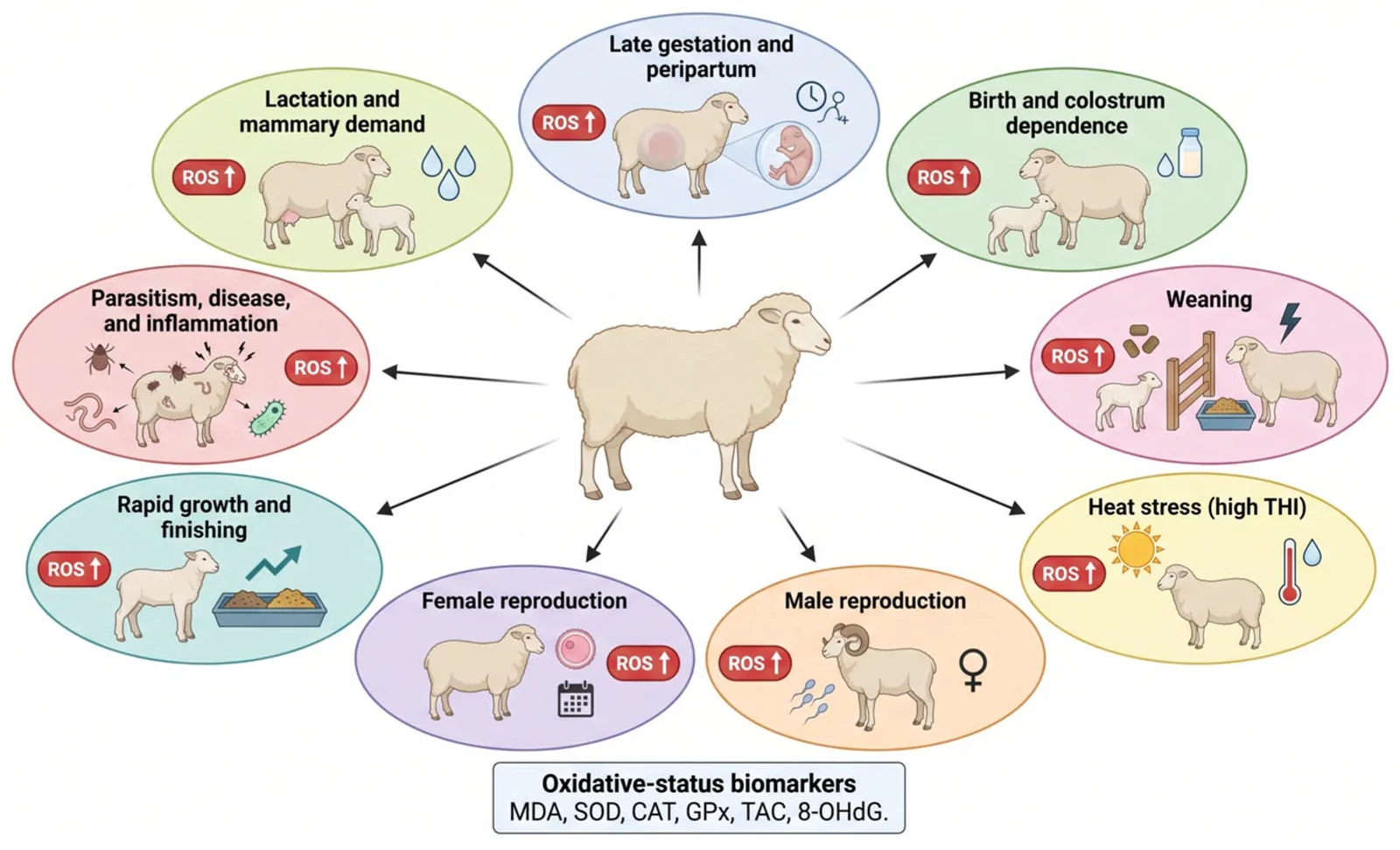
Physiological and environmental windows in which oxidative challenge may increase in sheep, together with commonly reported biomarkers. The magnitude and direction of change vary with tissue, timing, health status, and assay; the figure does not imply that every animal experiences oxidative stress at every listed stage. Arrows (↑) indicate an increase in reactive oxygen species (ROS) production or oxidative challenge during the corresponding physiological or environmental stage.

The transition from late gestation to early lactation is the most studied physiological window. Rising energy demand, variable feed intake, lipid mobilization, endocrine change, and inflammation can alter oxidant and antioxidant markers [8,18,19,20]. In some cohorts, MDA or total oxidant status increased while TAC or antioxidant-enzyme activities decreased near lambing; parity, breed, diet, health status, sampling time, and assay choice modified the pattern [18,19,20]. These findings support a period of increased oxidative challenge in susceptible ewes, but they do not establish a uniform acute oxidative episode in every animal. An overview of these physiological and environmental windows is presented in Figure 1.

At birth, the lamb moves into an oxygen-rich environment while endogenous defenses and passive transfer are still developing. Oxidant indices were higher in neonatal lambs that later died than in survivors in one observational study, but survival also depends on dystocia, birth weight, thermoregulation, infection, maternal care, and the amount and timing of colostrum intake [21]. Weaning, transport, rapid growth, and heat exposure can also disturb redox markers. A sheep heat-stress meta-analysis reported changes in intake, body weight, cortisol, selenium, lipid and protein oxidation, and antioxidant enzymes, with substantial heterogeneity [22]. Reproduction introduces additional contexts: physiological ROS participate in sperm capacitation and fertilization, whereas excessive ROS during semen processing or embryo culture can damage membranes and DNA [23,24]. Infection and parasitism increase oxidant production during immune activation, but host redox adaptation should not be conflated with a compound’s direct antimicrobial or anthelmintic action [25,26].

### 2.3. Measurement and Interpretation of Oxidative Status

Oxidative status can be assessed, but no single circulating surrogate is a definitive measure of whole-animal oxidative stress or Nrf2 activity. MDA and thiobarbituric acid-reactive substances (TBARS) are widely used indices of lipid oxidation, although TBARS has limited chemical specificity and is sensitive to sample handling. Protein carbonyls and 8-hydroxy-2′-deoxyguanosine provide information on protein and DNA oxidation, respectively [27]. SOD, CAT, and GPx activities describe components of antioxidant defense, but increases may reflect adaptation, inflammation, micronutrient status, or altered cell composition. TAC is influenced strongly by constituents such as uric acid and albumin, and results from different commercial kits are not necessarily comparable. A defensible interpretation therefore combines at least one damage marker, one defense marker, relevant clinical or production measures, and standardized sampling; pathway activation requires additional molecular evidence. The interpretation of commonly used redox markers across physiological and environmental windows is summarized in Table 1.

## 3. The Nrf2-Keap1-ARE Signaling Pathway

### 3.1. Core Architecture and Keap1-Dependent Regulation

**Figure 2:**
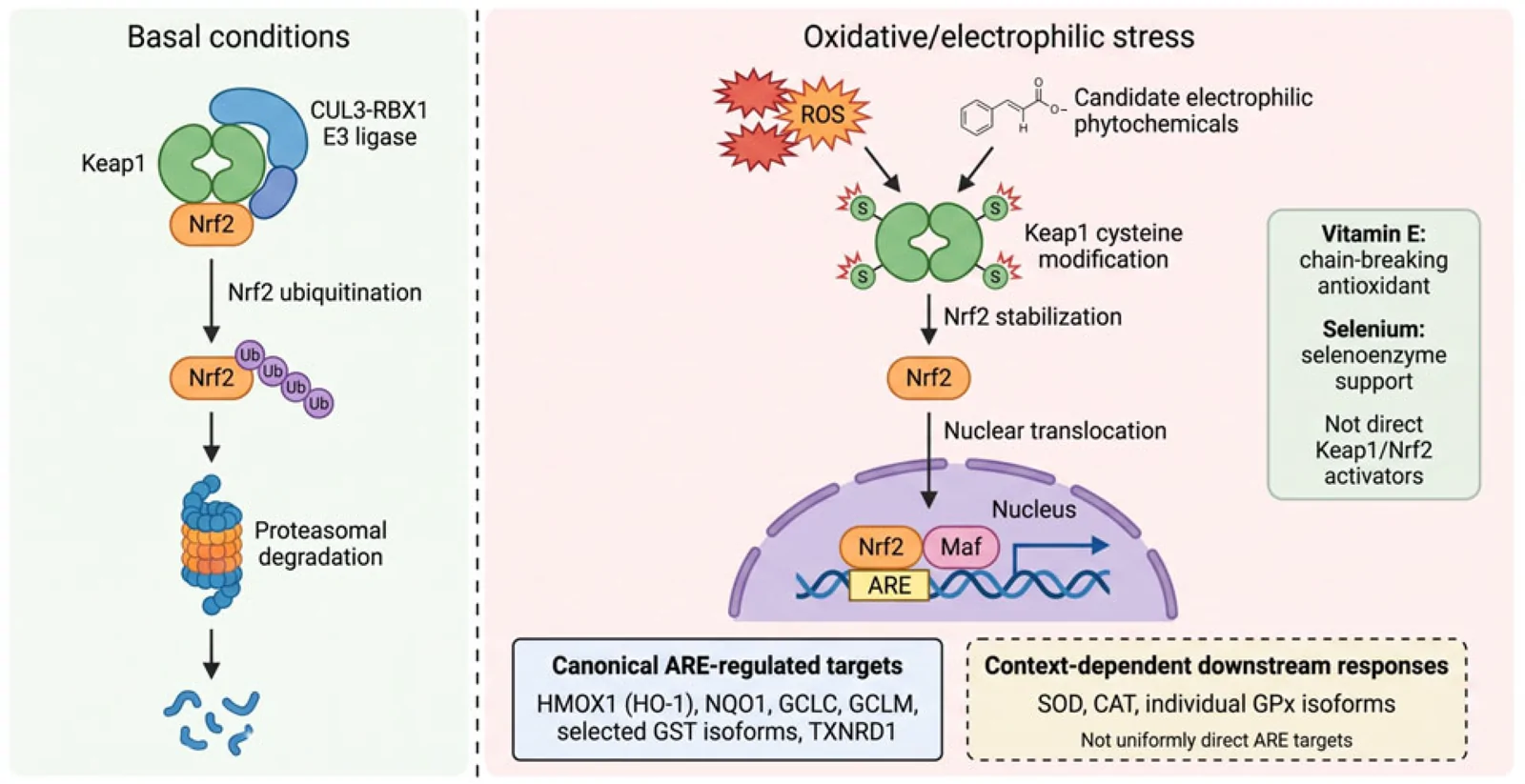
Simplified Keap1-dependent control of *Nrf2* under basal and electrophilic/oxidative conditions. Protein names are shown in roman type; gene symbols are italicized in the text and tables. Keap1-independent regulatory routes are described in Section 3.1.

*Nrf2*, encoded by *NFE2L2*, is a cap’n’collar basic leucine zipper transcription factor. Under basal conditions, Keap1 recruits Nrf2 to a Cullin-3/RING-box 1 E3 ubiquitin-ligase complex, promoting rapid ubiquitination and proteasomal turnover [10,11]. Electrophiles or oxidants modify sensor cysteines in *Keap1* and alter the geometry or efficiency of *Nrf2* ubiquitination. This de-repression mechanism allows newly synthesized Nrf2 to accumulate; it should not be described as a complete cessation of ubiquitin transfer in every context [10]. A schematic overview of this canonical pathway is shown in Figure 2.

Stabilized *Nrf2* can accumulate in the nucleus, heterodimerize with small Maf proteins, and bind *ARE* sequences to regulate cytoprotective genes [10,14]. *Nrf2* is also controlled independently of *Keap1* through pathways that include glycogen synthase kinase 3/β-TrCP, kinase-dependent phosphorylation, Bach1 competition, and p62-mediated autophagy signaling [11,32,33,34]. Consequently, an increase in *NFE2L2* transcript alone does not demonstrate Nrf2 stabilization, nuclear translocation, DNA binding, or transcriptional activity.

### 3.2. Cytoprotective Outputs

Well-supported Nrf2 outputs include genes involved in glutathione synthesis and recycling, thioredoxin/peroxiredoxin systems, NADPH regeneration, quinone reduction, glutathione conjugation, heme and iron metabolism, and xenobiotic defense [13,14,25,32]. By contrast, SOD, CAT, and individual GPx isoforms are not uniformly direct ARE targets across tissues and species. Their expression or activity may change downstream of Nrf2, through other transcription factors, through micronutrient supply, or through altered oxidative burden. They are therefore described here as Nrf2-related or downstream antioxidant readouts unless direct regulation has been demonstrated in the relevant model. Representative cytoprotective genes and the limitations of inferring direct Nrf2 regulation are summarized in Table 2.

### 3.3. Context-Dependent Crosstalk with Inflammation

Nrf2 and NF-κB interact through several mechanisms, including redox-sensitive signaling, competition for transcriptional co-regulators, HO-1-derived mediators, and changes in cellular metabolism [14,35]. Their relationship is not a universal on/off opposition: cell type, timing, stimulus, tissue redox state, and signaling intensity can produce parallel, sequential, or divergent responses. Therefore, concurrent increases in Nrf2-related markers and decreases in inflammatory mediators are biologically compatible but do not prove that Nrf2 caused the anti-inflammatory effect.

### 3.4. What Has Been Measured in Sheep

Two verified dietary studies provide the most direct ovine evidence. In 24 transition-period Hu ewes, rutin at 50 or 100 mg/kg body weight/day from 28 days prepartum to 28 days postpartum increased mammary *NFE2L2* and *HMOX1* mRNA and improved several oxidative, inflammatory, and apoptotic measures [36]. Total Nrf2 protein was not significantly altered, and *NQO1* mRNA did not change significantly; this was a transition feeding study, not an experimental mastitis model. In 32 Dorper × Han ewe lambs, dietary water extract of *Artemisia annua* at 0, 500, 1000, or 1500 mg/kg diet for 60 experimental days after a 15-day adaptation altered rumen-tissue expression of *Nrf2/Keap1*-related and antioxidant genes and improved rumen redox and immune indices, with 1000 mg/kg identified by the authors as the most favorable tested dose [37]. Because both studies relied mainly on expression and associated outcomes without Nrf2-specific perturbation, their evidence is pathway-associated rather than causal.

## 4. Dietary Antioxidants and Nrf2-Related Responses

### 4.1. Multiple Mechanisms, Evidence Boundaries, and Hormesis

Dietary antioxidants can act through overlapping mechanisms. Tocopherols can interrupt lipid-peroxidation chains in membranes, while some electrophilic phytochemicals or their metabolites may modify Keap1 or activate upstream kinases [11,38,39,40]. Polyphenols may also chelate metals, alter mitochondrial function, bind receptors, modify inflammatory signaling, or be converted by rumen microbes into bioactive metabolites [41,42,43,44]. Low plasma concentrations of a parent polyphenol therefore do not establish that Nrf2 induction is its predominant in vivo mechanism. Likewise, an increase in antioxidant-enzyme activity or a decrease in MDA is compatible with, but not diagnostic of, Nrf2 engagement.

**Figure 3:**
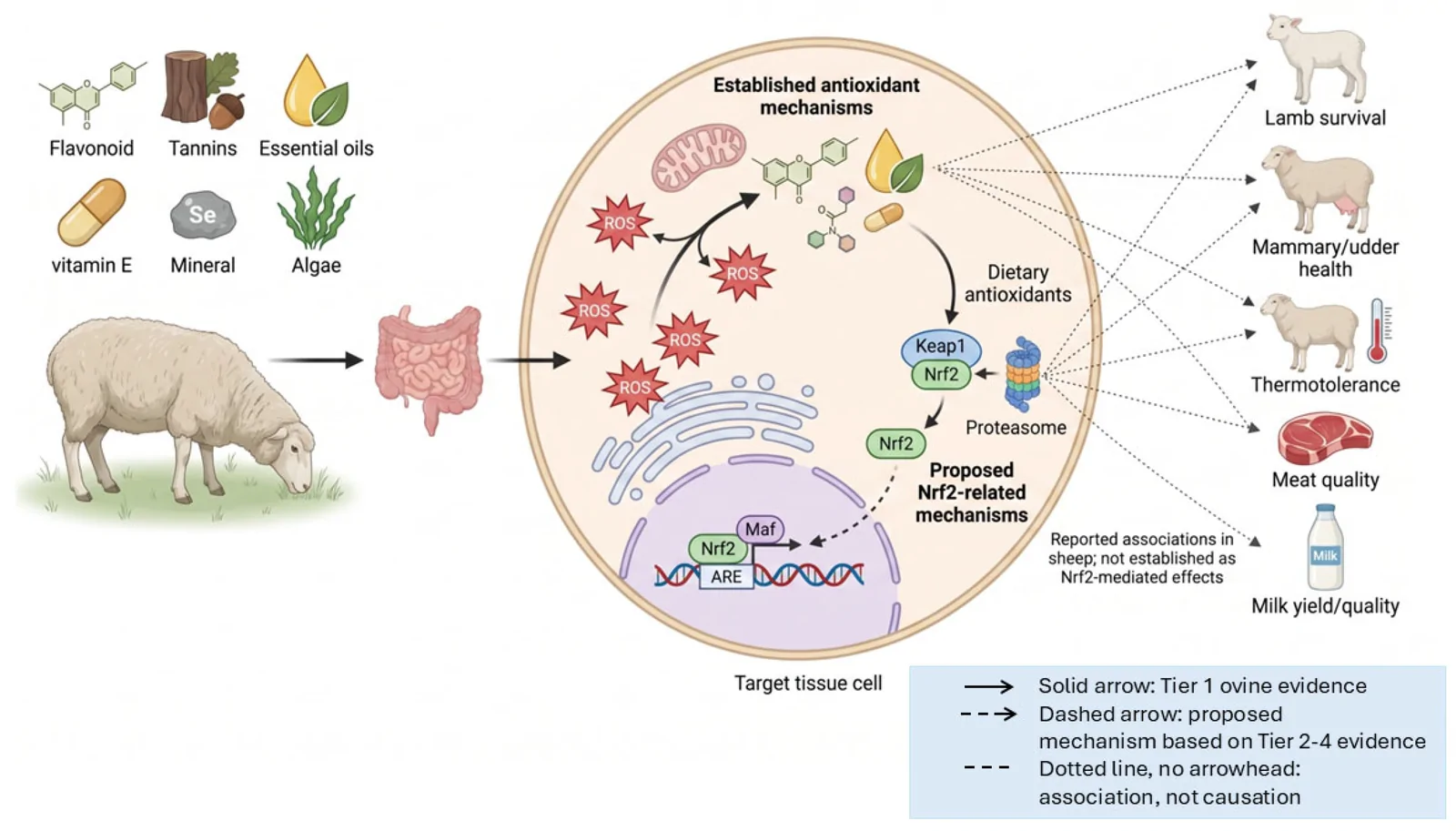
Conceptual routes by which dietary antioxidant classes may influence redox biology and animal outcomes. Solid interpretation should be limited to the evidence level described in Table 3 and Table 4. Vitamin E and selenium primarily support membrane protection and selenoenzyme function, respectively, and are not presented as direct Nrf2 activators. Several proposed Nrf2 mechanisms derive from non-ovine models; the figure does not imply that every class directly activates Nrf2 in sheep or that pathway-associated changes cause productivity outcomes.

The dose–response may be nonlinear. Mild electrophilic or oxidative exposure can trigger adaptive defenses, whereas excessive exposure can inhibit intake, disturb rumen fermentation, become pro-oxidant or cytotoxic, or create reductive stress [12,17,45,46]. Any claim of an “optimal” dose should therefore be restricted to the tested preparation, basal diet, breed, physiological state, exposure period, and outcomes. The practical safety considerations are developed in Section 4.9. Figure 3 summarizes how the main classes feed into this shared pathway.

### 4.2. Polyphenols and Flavonoids

Rutin provides the clearest ovine dietary example but supports a restrained interpretation: mammary *NFE2L2* and *HMOX1* transcripts increased, several antioxidant and inflammatory measures improved, total Nrf2 protein did not change significantly, and no causal pathway test was performed [36]. A standardized red–orange and lemon extract increased SOD, CAT, GPx, and TAC and decreased MDA, urinary 8-hydroxy-2′-deoxyguanosine, and inflammatory cytokines in lambs [27]. Because the citrus study did not measure Nrf2, it is Tier 2 evidence for an antioxidant and anti-inflammatory response, not direct pathway activation.

Resveratrol, luteolin, icariin, morin, and grape-seed proanthocyanidins have stronger Nrf2-related mechanistic support in pig, poultry, rodent, or cell models [42,47,48,49,50,51,52,53]. These studies identify plausible mechanisms relevant to livestock exposures but cannot establish efficacy, metabolism, tissue exposure, or dose in sheep. In ovine reproductive preparations, chlorogenic acid improved the redox profile of chilled ram semen [44], and dietary curcumin nano-micelles improved redox and some performance measures in heat-stressed lambs [54]; neither study demonstrated Nrf2 activation. Rumen metabolism may convert parent polyphenols into compounds with different absorption and biological activity, making parent-compound extrapolation particularly uncertain [43,55].

### 4.3. Condensed and Hydrolysable Tannins

Tannins are among the most extensively studied phytochemicals in sheep. Meta-analyses and controlled trials report changes in growth, nitrogen use, antioxidant indices, meat MDA or TBARS, metmyoglobin, fatty-acid profiles, and carcass traits, but effect sizes and directions vary by tannin structure, source, dose, basal diet, and animal condition. Chestnut tannins increased antioxidant-enzyme activities and improved selected growth or meat-quality outcomes in some lamb studies, and transcriptomic changes in *CAT* and *SOD1* were reported [56,57].

These transcripts are downstream redox readouts and do not by themselves establish direct Nrf2 activation. Condensed tannins from *Acacia mearnsii* or *Cistus ladanifer* have improved selected indices of meat oxidative stability in some studies [58,59]. Tannin intake must be interpreted against the total diet. There is no universal safe or effective inclusion rate because biological activity depends on molecular structure and background forage; many applied studies use approximately 10–40 g extract/kg diet dry matter, while the risk of reduced palatability, protein and mineral availability, fiber digestion, and growth generally increases as total biologically active tannins approach or exceed about 50 g/kg dry matter [45,60,61]. These values are context markers rather than prescriptions. Chemical characterization, basal-diet tannin analysis, intake monitoring, and dose-ranging are essential before field use.

### 4.4. Essential Oils, Terpenoids, and Aqueous Botanical Extracts

Essential oils contain volatile terpenoids and phenylpropanoids such as thymol, carvacrol, eugenol, and cinnamaldehyde. A small-ruminant meta-analysis reported average changes in antioxidant indices, rumen fermentation, performance, and product quality, but substantial between-study heterogeneity limits universal recommendations [62]. Oregano essential oil improved selected antioxidant and meat-storage outcomes in lambs [63]. These results are Tier 2 evidence because Nrf2 was not measured. Essential oils should be described as potential components of antibiotic-reduction strategies, not as leading substitutes for antibiotics; antimicrobial efficacy, resistance prevention, animal welfare, and veterinary oversight remain separate requirements [26,33,64].

The *Artemisia annua* intervention was a water extract, not an essential oil, and is therefore treated separately. Its rumen-tissue gene-expression data constitute Tier 1 pathway-associated ovine evidence [37]. Encapsulated oregano, cinnamon, and clove oils increased Nrf2-related transcripts in broilers [65], but this Tier 4 evidence does not establish the same response in the rumen or other sheep tissues. Volatile oils can also alter intake, rumen microbial ecology, fermentation, and sensory properties of meat or milk; dose, encapsulation, withdrawal conditions, and residue or off-flavor monitoring should be reported.

### 4.5. Vitamin E and C

Vitamin E is primarily a lipid-phase, chain-breaking antioxidant and remains a reference intervention for protecting biological membranes and animal products [5,39]. Its effects should not be labeled as direct Nrf2 activation without pathway-specific evidence. Vitamin E and selenium interact functionally: deficiency can contribute to nutritional muscular dystrophy in lambs, while supplementation can improve antioxidant status when supply is inadequate [66]. Evidence from dairy cows must be labeled bovine and cannot be transferred automatically to ewes [67]. Adult sheep synthesize vitamin C, and ruminal degradation limits routine oral use; protected forms or short-term stress applications require direct ovine evaluation.

### 4.6. Selenium and Trace Minerals

Selenium supports the catalytic function of GPx and thioredoxin reductases, several components of which lie within Nrf2-regulated networks [15,16]. Selenium supply can therefore constrain downstream antioxidant capacity, but supplementation is not equivalent to direct Nrf2 activation. Maternal selenium increased selenium status and selected antioxidant-enzyme activities in ewes and lambs [68], and selenium with vitamin E improved mammary-health measures, including lower mastitis incidence, in one ewe study [69]. Manganese and zinc support antioxidant enzymes and other cellular functions, but enzyme activity changes do not identify an Nrf2 mechanism [70,71]. Because selenium has a narrow margin between deficiency and toxicity, supplementation should follow regional feed regulations, baseline forage and blood assessment, total-diet calculation, and veterinary or qualified nutritionist oversight. Similar care is needed for zinc and manganese to avoid toxicity, mineral antagonism, and environmental loading.

### 4.7. Carotenoids, Algae, and Agro-Industrial By-Products

Carotenoid-rich feeds, grape residues, seaweeds, and spirulina can contribute pigments, polyphenols, vitamins, minerals, and other bioactives. In sheep, grape residue flour, *Sargassum latifolium*, and spirulina-based interventions improved selected antioxidant, inflammatory, physiological, or milk-quality indices under heat stress [72,73,74]. These are Tier 2 outcomes unless Nrf2-specific tissue measurements are included. The active constituent cannot be inferred from a complex feed ingredient, and benefits may arise through nutrient supply, rumen effects, pigmentation, inflammation control, or multiple combined mechanisms. Contaminants, iodine or mineral excess, batch composition, palatability, and product residues should be considered for algae and by-products.

### 4.8. Evidence Map for Dietary Antioxidants in Sheep

Table 3 summarizes the dietary antioxidant classes evaluated in sheep, their reported outcomes, and the strength of evidence for Nrf2 engagement. Table 4 focuses on the two ovine dietary studies that measured Nrf2-related molecular endpoints and reports their animal models, doses, tissues, pathway readouts, outcomes, and mechanistic limitations. Together, the evidence map shows that most ovine studies remain Tier 2, whereas only the rutin and *Artemisia annua* studies meet Tier 1 criteria, and neither establishes causal Nrf2 activation.

**Table 3:** Dietary antioxidant classes in sheep, reported outcomes, and evidence level for Nrf2 engagement.

Class/Intervention	Ovine Evidence and Reported Outcomes	Nrf2 Interpretation	Refs.
Rutin	Transition-period ewe feeding; mammary redox, inflammation, apoptosis, and gene expression	Tier 1; pathway-associated, not causal; total Nrf2 protein unchanged	[36]
Citrus polyphenol extract	Lamb feeding; antioxidant enzymes, damage markers, and cytokines	Tier 2; no Nrf2 assay	[27]
Tannins	Sheep feeding; variable growth, rumen, redox, carcass, and meat-stability outcomes	Tier 2; downstream genes/enzymes do not establish Nrf2 activation 45,56,61,75,76	[45,56,57,58,59,60,61,75,76]
Essential oils	Small-ruminant and lamb feeding; variable rumen, performance, redox, and product outcomes	Tier 2 in sheep; Nrf2 evidence mainly non-ovine 63,63,65	[62,63,65]
*A. annua* water extract	Lamb feeding; rumen immune, fermentation, redox, and gene-expression outcomes	Tier 1; pathway-associated, not causal	[37]
Vitamin E	Sheep feeding; membrane protection, deficiency prevention, heat and product-stability outcomes	Tier 2; primarily direct lipid-phase antioxidant	[23,26,39]
Selenium/trace minerals	Ewe/lamb feeding; mineral status and antioxidant-enzyme outcomes	Tier 2; supports enzyme function, not direct activation	[16,68,69,70,71]
Curcumin nano-micelles	Heat-stressed lamb feeding; redox and performance outcomes	Tier 2	[54]
Algae/by-products	Heat-stressed sheep; redox, immune, physiological, or milk-quality outcomes	Tier 2; complex-ingredient mechanisms unresolved	[72,73,74]
Reproductive antioxidants	Mostly semen extender, cryopreservation, embryo culture, or cell studies	Not dietary Nrf2 evidence; see Section 5.5	[24,30,44,77]

Abbreviations: Nrf2, nuclear factor erythroid 2-related factor 2; Refs., references. Tier 1–4 evidence levels are defined in Section 1.

**Table 4:** Direct dietary studies measuring Nrf2-related molecular readouts in sheep.

Intervention and Ovine Model	Dose and Duration	Pathway-Related Readouts	Main Outcomes and Evidence Interpretation	Ref.
Rutin; 24 transition-period Hu ewes; mammary tissue sampled 28 d postpartum	0, 50, or 100 mg/kg BW/day; day −28 to +28 relative to parturition	Mammary *NFE2L2* and *HMOX1* mRNA increased; *NQO1* mRNA not significant; total Nrf2 protein not significant	Redox, inflammatory, and apoptotic markers improved. Tier 1, pathway-associated; no nuclear localization, ARE assay, or Nrf2 perturbation. Not a mastitis model.	[36]
*Artemisia annua* water extract; 32 Dorper × Han ewe lambs; rumen tissue	0, 500, 1000, or 1500 mg/kg diet; 15 d adaptation + 60 d trial	Rumen Nrf2/Keap1-related and antioxidant-gene expression; MDA and enzyme indices	Rumen immune, fermentation, redox, and microbial outcomes changed; 1000 mg/kg was most favorable among tested doses. Tier 1, pathway-associated; expression-based and non-causal.	[37]

Abbreviations: ARE, antioxidant response element; BW, body weight; d, day(s); *HMOX1*, heme oxygenase-1 (gene); MDA, malondialdehyde; mRNA, messenger ribonucleic acid; *NFE2L2*, nuclear factor erythroid 2-like 2 (gene encoding Nrf2); *NQO1*, NAD(P)H quinone oxidoreductase 1; Nrf2, nuclear factor erythroid 2-related factor 2; Ref., reference.

### 4.9. Practical Selection, Dose, and Safety

A supplement should be selected only after defining the production stage, plausible oxidative challenge, basal-diet adequacy, target outcome, and measurement plan. Product chemistry, bioavailability, rumen transformation, cost, legal limits, and risk of adverse effects are at least as important as the proposed Nrf2 mechanism. The framework in Table 5 is deliberately conditional: it identifies where evidence is stronger, what should be monitored, and when a supplement should be reconsidered. It is not a universal dose schedule.

**Table 5:** Decision framework for antioxidant use in sheep production.

Context and Goal	Candidate Strategy	Evidence Strength	Monitor	Main Cautions
Documented vitamin E/Se inadequacy; transition or heat risk	Correct the measured nutrient gap within local regulations	Moderate for redox/deficiency outcomes; Nrf2 mechanism unproven	Total diet, blood/forage status, GPx, clinical signs	Selenium toxicity; mineral interactions; bovine data are not ovine data
Heat exposure with reduced intake or oxidative markers	Dietary antioxidant package plus shade, water, ventilation, and energy management	Moderate for redox/physiological outcomes; variable growth	THI, intake, temperature, respiration, damage + defense markers	Do not replace environmental control; avoid supranutritional assumptions
Meat oxidative stability	Vitamin E or chemically characterized tannin/plant source	Moderate but heterogeneous	Diet intake, tissue deposition, MDA/TBARS, color, sensory/storage outcomes	Tannin antinutrition; oil off-flavors; storage and packaging effects
Rumen or gut target	Characterized tannin, essential oil, or aqueous botanical preparation	Limited to moderate and preparation-specific	Intake, digestibility, fermentation, microbiota, health endpoints	Microbial adaptation, reduced fiber digestion, palatability, antimicrobial claims
Reproduction	Correct nutritional deficiency; distinguish feeding from semen/embryo additives	Limited dietary evidence	Fertility endpoints, semen/embryo measures, exposure and safety	Ex vivo benefits cannot justify feeding recommendations; physiological ROS are required

Abbreviations: GPx, glutathione peroxidase; MDA, malondialdehyde; Se, selenium; TBARS, thiobarbituric acid-reactive substances; THI, temperature–humidity index.

## 5. Implications for Sheep Health

### 5.1. Transition Period and Mammary Health

The transition period combines metabolic, endocrine, immune, and management changes, making it a logical target for nutritional support but a difficult setting for causal inference. Rutin altered mammary redox, inflammatory, apoptotic, and Nrf2-related transcript measures in healthy transition-period ewes [36]. This study did not model mastitis and did not prove that Nrf2 mediated the tissue effects. Vitamin E/selenium evidence from dairy cows is labeled as bovine [67], whereas ewe studies support selected antioxidant or mammary-health outcomes [68,69]. Grape residue flour lowered somatic cell count and lipid-peroxidation indices in heat-stressed dairy ewes without a consistent milk-yield effect [72]. These findings support further targeted trials, not a general claim that Nrf2 activation prevents transition disease.

### 5.2. Neonatal Status and Passive Transfer

Neonatal oxidant indices are associated with early health and survival, and colostrum supplies immunoglobulins, nutrients, vitamins, and antioxidant components [21]. Maternal selenium and organic manganese interventions changed selected antioxidant-enzyme and passive-transfer measures in ewes and lambs [68,70]. Nevertheless, available studies do not isolate Nrf2 as the mediator or establish that antioxidant supplementation independently improves lamb survival. Dystocia, birth weight, litter size, colostrum timing and volume, hypothermia, infection, maternal behavior, and management remain major confounders. Claims are therefore limited to redox and passive-transfer outcomes unless mortality was prospectively powered and directly measured.

### 5.3. Gastrointestinal Integrity, Immunity and Parasite Challenge

Poorly absorbed phytochemicals and their microbial metabolites may reach higher concentrations in the gastrointestinal tract than in peripheral tissues [43,55,61]. The *A. annua* water-extract study associated rumen Nrf2-related gene expression with antioxidant, immune, fermentation, and microbial changes in lambs [37]. Non-ruminant studies provide mechanistic support for interactions among Nrf2, epithelial barriers, and inflammatory signaling [41,48], but rumen physiology and metabolism limit direct extrapolation. Tannins and essential oils may also affect parasites directly; those anthelmintic effects should be separated from host Nrf2-related responses. Reduced fecal egg count or parasite viability does not demonstrate Nrf2-mediated immunity [31].

### 5.4. Reproduction: Dietary, Ex Vivo, and In Vitro Evidence

The reproductive literature contains biologically distinct interventions. Dietary studies in live sheep are limited and include mixed results from tannin preparations and antioxidant packages [78,79]. Most favorable evidence cited in this field concerns direct addition to semen extenders or embryo-culture media: chlorogenic acid in chilled ram semen [44], MitoPBN and MitoQ during cryopreservation [80,81], lavender extract and Trolox in semen-processing protocols [24,77], and resveratrol in vitrified sheep embryos [30]. These experiments can improve post-storage or in vitro endpoints but do not test dietary exposure, rumen metabolism, tissue bioavailability, or feeding efficacy. The sulforaphane study measured NRF2/ARE-related responses in human granulosa cells, not in sheep and not after dietary administration [82]. It is retained only as Tier 4 mechanistic evidence. The previous discussion of the melatonin receptor 1A polymorphism was removed because it did not establish a direct connection between antioxidant intervention, Nrf2 signaling, and reproductive outcome. Future ovine feeding trials should measure reproductive endpoints prospectively and separate male, female, semen-processing, embryo-culture, and whole-animal questions.

### 5.5. Thermotolerance

Antioxidant supplements can be evaluated as adjuncts to, not replacements for, heat-abatement management. Vitamin E/selenium, curcumin nano-micelles, essential-oil blends, fish oil with vitamin E and selenium, seaweed, and spirulina have improved selected oxidative, immune, physiological, or performance measures in heat-exposed sheep [29,54,73,74,83,84]. The antioxidant response is more consistent than the growth response, and none of these outcomes proves Nrf2 mediation. Benefits are likely to depend on heat load, diet adequacy, supplement formulation, intake, acclimation, and baseline health.

## 6. Implications for Productivity and Product Quality

### 6.1. Growth and Feed Efficiency

Growth and feed-efficiency responses are heterogeneous. Meta-analyses of tannins or essential oils report modest average effects with substantial dependence on source, dose, diet, and production conditions [62,75,76]. Some heat-stress studies improved physiological or redox measures without significant growth gains, whereas others reported concurrent performance improvements [83,84]. The defensible conclusion is that antioxidant interventions may help preserve performance when a specific oxidative, nutritional, or environmental constraint is present; they should not be presented as direct growth promoters or as proven consequences of Nrf2 activation.

### 6.2. Meat Oxidative Stability, Color and Shelf Life

Meat-quality outcomes are often more consistently observed than live-animal productivity outcomes, but the evidence has not been formally graded and remains heterogeneous. Tannin meta-analysis and individual chestnut, oregano, *Cistus ladanifer*, and vitamin E studies report improvements in one or more measures of lipid oxidation, pigment stability, shelf life, or sensory-related quality [39,56,59,63,75]. The magnitude depends on tissue deposition, antioxidant source and dose, basal diet, slaughter conditions, packaging, oxygen exposure, light, storage temperature, and storage duration. These endpoints should therefore be described as frequently favorable under tested conditions, not universally reliable.

### 6.3. Milk Yield, Quality and Fatty-Acid Profile

In dairy ewes, selected supplements have altered somatic cell count, lipid peroxidation, milk solids, or feed efficiency, while milk-yield responses have been inconsistent [62,72]. Tannins and essential oils can also modify rumen biohydrogenation and the fatty-acid profile of milk or meat [57,76].

Greater deposition of polyunsaturated fatty acids is not automatically a net benefit because these lipids are more susceptible to oxidation; the outcome depends on the degree of enrichment, concomitant antioxidant deposition, processing, packaging, and storage. Claims should therefore specify both nutritional composition and oxidative stability rather than treating polyunsaturated-fat enrichment alone as improvement.

## 7. Knowledge Gaps and Research Priorities

The first priority is causal, tissue-specific validation. Rutin and *A. annua* provide useful pathway-associated ovine data [85], but expression assays cannot establish Nrf2 dependence. Stronger studies should measure nuclear Nrf2, ARE binding or reporter activity where feasible, canonical target transcripts and proteins, enzyme function, and tissue redox outcomes. Nrf2-specific perturbation, validated pharmacological inhibition, genetic approaches, or orthogonal target-engagement methods are needed to distinguish causation from correlation. Applying these methods in sheep involves sampling, assay-validation, isoform, antibody, and intervention-design challenges; it is not merely a matter of using existing technology.

The second priority is exposure-aware dose–response work. Studies should characterize the supplement chemically, quantify basal-diet intake, measure parent compounds and rumen-derived metabolites where possible, and relate tissue exposure to both benefit and toxicity. Designs should include enough dose levels to detect biphasic responses and should monitor intake, digestibility, rumen fermentation, mineral balance, liver and kidney safety, product residues or off-flavors, and evidence of reductive stress. An “optimal” dose should be defined only for the tested preparation and context.

The third priority is better outcome and biomarker standardization. At least one oxidative-damage marker and one antioxidant-defense marker should be paired with clinical, immune, reproductive, productivity, or product-quality endpoints. Pre-analytical handling, assay platform, timing, tissue, and reporting units should be standardized; TAC and TBARS results from different kits should not be assumed equivalent. Trials should be powered for the claimed outcome and should report null findings. Breed, parity, litter size, health status, climate, diet, housing, and management should be controlled or reported.

Finally, future synthesis should preserve evidence boundaries. Other-ruminant and monogastric experiments are valuable for hypothesis generation, but rumen metabolism, tissue exposure, and species-specific physiology require ovine confirmation. Reproductive additives used directly in semen or embryo media should not be interpreted as dietary evidence. Product-quality claims should integrate storage conditions, sensory outcomes, and fatty-acid oxidation. These improvements would allow future systematic review and quantitative grading of benefit, risk, and practical value.

## 8. Conclusions

Nrf2 provides a useful framework for understanding adaptive redox responses in sheep, but the dietary evidence is much narrower than the mechanistic literature implies. Two verified ovine feeding studies reported Nrf2-related gene-expression changes, and neither demonstrated causal pathway activation. Most sheep studies support effects on oxidative-status markers, health-related measures, or product stability without identifying the mechanism. Dietary antioxidants may act through Nrf2-related signaling, direct radical interception, nutrient-cofactor supply, rumen and microbial metabolism, mitochondrial effects, receptor pathways, and inflammation control. Benefits to growth, fertility, milk yield, and survival remain variable or insufficiently isolated from confounding factors. Therefore, antioxidant feeding should be matched to a defined nutritional or environmental need, evaluated with multiple outcomes, and constrained by preparation-specific dose and safety data. Causal pathway assays, exposure measurements, and well-powered ovine trials are required before Nrf2-targeted nutrition can be considered a dependable productivity strategy.

## Figures and Tables

**Table 1:** Redox-challenge windows, commonly used measurements, and interpretation limits in sheep.

Window/Driver	Commonly Reported Pattern	Measures	Interpretation and Representative Evidence
Late gestation and early lactation	Higher oxidant or inflammatory indices and lower antioxidant reserve in some cohorts	MDA/TBARS, TOS, TAC/TAS, SOD, CAT, GPx, haptoglobin	Depends on parity, breed, diet, health, and sampling; [8,18,19,20]
Birth and neonatal period	High oxidant indices in some lambs, followed by adaptation	TOS, TAS, oxidative-stress index, IgG	Association with mortality does not establish that antioxidant feeding improves survival; [21]
Weaning and transport	Transient oxidative and inflammatory responses	TBARS, ceruloplasmin, CAT, cortisol	Timing and concurrent nutritional or infectious stress are important; [28]
Heat exposure	Variable changes in MDA, protein oxidation, enzymes, cortisol, and intake	THI, rectal temperature, respiration, MDA, protein carbonyls, SOD, Se	Meta-analytic pattern is heterogeneous; [22,29]
Reproductive handling	Excess ROS during semen cooling/freezing or embryo culture	ROS, MDA, TAC, motility, DNA fragmentation, embryo development	Mostly ex vivo/in vitro and not evidence for dietary Nrf2 activation; [23,24,30]
Disease and parasitism	Respiratory-burst ROS and inflammatory signaling	Damage markers, cytokines, cell counts, clinical burden	Direct antimicrobial/anthelmintic action must be separated from host Nrf2 effects; [25,31]

Abbreviations: CAT, catalase; DNA, deoxyribonucleic acid; GPx, glutathione peroxidase; IgG, immunoglobulin G; MDA, malondialdehyde; Nrf2, nuclear factor erythroid 2-related factor 2; ROS, reactive oxygen species; SOD, superoxide dismutase; TAC/TAS, total antioxidant capacity/status; TBARS, thiobarbituric acid-reactive substances; THI, temperature–humidity index; TOS, total oxidant status.

**Table 2:** Representative genes directly or indirectly regulated within Nrf2-related cytoprotective networks.

Gene(s)	Protein/System	Function	Interpretive Note
*HMOX1*	Heme oxygenase-1	Heme catabolism; antioxidant and immunomodulatory products	Widely used pathway-related target
*NQO*1	NAD(P)H quinone oxidoreductase 1	Two-electron quinone reduction	Canonical ARE-regulated target
*GCLC/GCLM*	Glutamate-cysteine ligase	Rate-limiting GSH synthesis	Canonical Nrf2-regulated system
*GSR*	Glutathione reductase	NADPH-dependent GSSG reduction	Supports glutathione recycling
*GSTs*	Glutathione S-transferases	Electrophile conjugation	Multiple isoforms are ARE-regulated
*TXN/TXNRD1/PRDXs*	Thioredoxin, thioredoxin reductase, peroxiredoxins	Protein-thiol and peroxide control	Links Nrf2 to thiol buffering
*SOD1/SOD2, CAT, GPXs*	Antioxidant enzymes	Superoxide and peroxide removal	Regulation is tissue- and isoform-dependent; activity alone is not proof of Nrf2 activation

Abbreviations: *CAT*, catalase; *GCLC/GCLM*, glutamate-cysteine ligase catalytic/modifier subunit; *GPXs*, glutathione peroxidases; GSH, reduced glutathione; *GSR*, glutathione reductase; GSSG, oxidized glutathione; *GSTs*, glutathione S-transferases; *HMOX1*, heme oxygenase-1 (gene); NADPH, nicotinamide adenine dinucleotide phosphate; *NQO1*, NAD(P)H quinone oxidoreductase 1; Nrf2, nuclear factor erythroid 2-related factor 2; *PRDXs*, peroxiredoxins; *SOD1/SOD2*, superoxide dismutase 1/2; *TXN*, thioredoxin; *TXNRD1*, thioredoxin reductase 1.

## Data Availability

No new data were created or analyzed in this study. Data sharing is not applicable to this article.
